# Targeting the *Helicobacter pylori*–Gastric Cancer Axis with Nanomaterials: From Infection Control to Tumor-Directed Therapy

**DOI:** 10.3390/molecules31213924

**Published:** 2026-11-01

**Authors:** Patrycja Jaroniek, Maria Połomska, Magdalena Chmiela, Weronika Gonciarz

**Affiliations:** 1 Department of Immunology and Infectious Biology, Institute of Microbiology, Biotechnology and Immunology, Faculty of Biology and Environmental Protection, University of Lodz, 91-278 Lodz, Poland; patrycja.jaroniek@edu.uni.lodz.pl (P.J.); maria.polomska@edu.uni.lodz.pl (M.P.); magdalena.chmiela@biol.uni.lodz.pl (M.C.); 2 BioMedChem Doctoral School of the University of Lodz and Lodz Institutes of the Polish Academy of Sciences, University of Lodz, 91-278 Lodz, Poland

**Keywords:** *Helicobacter pylori*, gastric cancer, nanomaterials, nanoparticles, drug delivery, chitosan, poly(lactic acid), tumor microenvironment, mucosal immunity, infection-associated models

## Abstract

*Helicobacter pylori* (*H. pylori*) infection promotes persistent mucosal inflammation, oxidative stress, epithelial injury, immune dysregulation, and progressive remodeling of the gastric microenvironment, thereby increasing the risk of gastric cancer (GC). Although *H. pylori* eradication and GC therapy are usually treated as separate clinical problems, they are linked by a continuum of pathogenic processes. Nanomaterial-based therapeutic systems may help overcome different barriers on this axis by improving drug stability and delivery, facilitating transport through mucus and biofilms, and enabling host-directed immunomodulation. This review focuses on polymeric, lipid-based, inorganic, hybrid, and stimuli-responsive approaches relevant to the management of *H. pylori* infection and the treatment of GC, emphasizing how carrier design can be adapted to different disease stages. Selected chitosan-based particles and functionalized poly(lactic acid) systems are discussed as examples of stage-adapted delivery strategies across the infection–cancer continuum. This review also describes three-dimensional and infection-associated GC models as tools for assessing nanoparticle penetration and therapeutic responses under more physiologically relevant conditions. Stage-adapted interventions are considered as distinct or potentially sequential approaches, rather than as a single validated platform combining bacterial eradication, inflammation control, mucosal repair, and anticancer therapy.

## 1. Introduction

### 1.1. From Helicobacter Pylori Infection and Inflammation to Gastric Cancer

Infection with the Gram-negative bacteria *Helicobacter pylori* (*H. pylori*) most commonly begins in early childhood, when they colonize the gastric mucus layer, particularly near the epithelial surface, and may persist throughout life. Approximately half of the global population is infected. *H. pylori* is capable of persistent colonization of the stomach due to numerous virulence factors, including urease, flagella, and outer membrane proteins that facilitate bacterial adhesion. Other virulence factors contribute to gastric tissue damage and the development of an inflammatory response [1,2]. Infection may lead to chronic gastritis and gastric or duodenal ulcers. Furthermore, gastric cancer (GC) and mucosa-associated lymphoid tissue (MALT) lymphoma are associated with chronic *H. pylori* infection, which can increase the production of pro-inflammatory cytokines, including interleukins (ILs), and the chemokine regulated on activation, normal T cell expressed and secreted (RANTES). Inflammatory mediators, particularly those involved in nuclear factor-κB (NF-κB) signaling, contribute to gastric pathology and play important roles in the development of gastritis and GC; however, the underlying molecular mechanisms are not fully understood [1,2].

Dysregulation of signaling pathways involved in cell proliferation, survival, and differentiation—including Janus kinase/signal transducer and activator of transcription (JAK/STAT), NF-κB, Wnt/β-catenin, mitogen-activated protein kinase (MAPK), Hippo, and phosphoinositide 3-kinase/protein kinase B (PI3K/Akt)—may contribute to *H. pylori*-associated carcinogenesis [3]. Infection with *H. pylori* has been classified by the International Agency for Research on Cancer (IARC) as carcinogenic to humans (Group 1) [4]. Among its virulence factors, cytotoxin-associated gene A (CagA) protein is particularly important in gastric carcinogenesis. CagA is delivered into gastric epithelial cells through the type IV secretion system and is subsequently phosphorylated at glutamic acid–proline–isoleucine–tyrosine–alanine (EPIYA) motifs. The EPIYA-A and EPIYA-B segments are widespread, whereas EPIYA-C predominates in Western-type CagA and EPIYA-D in East Asian-type CagA. Phosphorylated EPIYA-C or EPIYA-D segments bind to and dysregulate Src homology 2 domain-containing protein tyrosine phosphatase 2 (SHP2), resulting in abnormal extracellular signal-regulated kinase (ERK)/MAPK signaling, altered cell polarity, cytoskeletal remodeling, and increased cell motility. These effects contribute to the oncogenic potential of CagA-positive strains [3].

Treatment of GC may involve surgical resection in combination with systemic therapies, including chemotherapy and immunotherapy, depending on disease stage. However, late-stage diagnosis, tumor location, and rapid development of drug resistance in GC cells are key factors associated with disease recurrence [5]. Because *H. pylori* infection increases the risk of GC, eradication of active infection is recommended to reduce subsequent cancer risk [6]. The limited therapeutic efficacy of currently used anti-*H. pylori* therapies, partly attributable to resistance of *H. pylori* strains to commonly used antibiotics, particularly clarithromycin, metronidazole, and levofloxacin, poses a significant clinical challenge and underscores the need for alternative therapeutic approaches [6]. Experimental alternatives include plant-derived preparations, such as *Salvia cadmica* extracts formulated in polymeric microparticles, and host-directed approaches involving the vaccine strain *Mycobacterium bovis* Bacillus Calmette–Guérin (BCG); these strategies have shown immunomodulatory or anti-colonization potential in preclinical models but are not established substitutes for antibiotic eradication [7,8,9,10]. Nanotechnology-based approaches are being investigated to improve treatment efficacy.

Nanomaterial-based approaches have emerged as promising ways to address some limitations of conventional drug administration. Their physicochemical properties and drug-loading characteristics can be tailored to provide controlled, sustained, or targeted release. Moreover, nanoformulations may improve drug stability, bioavailability, and efficacy while reducing adverse effects. These advantages provide a rationale for exploring nanomaterial-based antibacterial, immunomodulatory, and anticancer strategies according to the disease stage [11,12,13].

The *H. pylori*–gastric cancer continuum encompasses distinct biological processes: persistent bacterial colonization and mucosal injury, potentially persistent premalignant tissue remodeling, and, in patients who develop cancer, tumor-specific survival and treatment-resistance mechanisms. These processes are connected by disease pathogenesis but do not constitute a single therapeutic target. Accordingly, nanomaterial-based interventions must be assessed according to the relevant disease stage, the relevant biological barrier, and the strength of the available experimental evidence.

### 1.2. Stage-Specific Therapeutic Targets and the Infection–Cancer Evidence Framework

The infection–cancer axis does not imply that every patient with *H. pylori* infection will progress to GC or that all therapeutic approaches must simultaneously target infection and malignancy. Rather, it provides a framework for identifying the dominant, potentially overlapping pathogenic mechanisms at different disease stages. Active infection, premalignant gastric lesions, and established GC require different therapeutic endpoints; importantly, advanced atrophy or intestinal metaplasia may persist after successful bacterial eradication [14,15].

During active *H. pylori* infection, viable bacteria within gastric mucus, adherent communities, biofilms, and potentially intracellular reservoirs are the primary therapeutic targets [16,17,18]. Locally administered particulate systems must therefore balance gastric stability and retention with access to the bacterial niche, and antibacterial activity should be distinguished from immunomodulatory or mucosal-protective effects. Relevant evidence includes changes in bacterial burden, biofilm viability, colonization, and infection-associated tissue injury. A reduction in bacterial load or inflammatory markers in a preclinical model, however, does not by itself demonstrate prevention of gastric cancer.

At the precancerous stage, gastric atrophy and intestinal metaplasia can coexist with ongoing infection or persist after eradication. Therapeutic targets therefore extend beyond pathogen elimination to include altered epithelial differentiation, oxidative and inflammatory signaling, impaired repair, and stromal remodeling. Carrier designs may require epithelial localization, controlled release at inflamed or oxidatively stressed sites, and minimal interference with mucosal regeneration. In this setting, studies that assess bacterial clearance alongside premalignant histological or molecular changes offer a more direct mechanistic link across the infection–cancer axis than studies measuring antibacterial activity alone [19]. Nevertheless, improvements in surrogate markers of metaplasia do not establish long-term cancer prevention.

In established GC, the major delivery barriers and therapeutic goals shift toward tumor heterogeneity, stromal barriers, intracellular drug exposure, and resistance to anticancer treatment. Nanocarriers studied exclusively in cell lines, spheroids, or tumor-bearing animals provide evidence for tumor-directed activity, not necessarily for effects on *H. pylori* infection or infection-driven carcinogenesis [20,21]. Exposing tumor models to defined bacterial components can reveal selected infection-associated responses, but remains distinct from chronic live infection and from immune-competent infection–tumor models [22]. This review therefore separates pathogen-directed evidence, studies that experimentally bridge infection and premalignant changes, and general gastric cancer nanotherapies; their possible integration is considered a future modular or sequential strategy rather than a validated single-treatment concept.

## 2. Biological Barriers to Nanomaterial-Based Therapy in the Stomach and Strategies to Overcome Them

### 2.1. Oral and Local Therapy: Gastric Mucus, Biofilms, and Epithelial Access

The stomach is a demanding site for local drug delivery because a carrier must remain functional despite low and fluctuating pH, digestive enzymes, mechanical mixing, variable gastric emptying, and continuous mucus turnover [16,23,24]. These constraints are important for *H. pylori*-directed therapy. Most bacteria are found in the mucus layer near the epithelial surface [16]. Gastric mucus contains a marked pH gradient, with strongly acidic conditions toward the lumen and a substantially less acidic environment near the epithelium, where *H. pylori* establish persistent colonization [16,23]. A formulation that is stable in the lumen must therefore still release or expose its active component at the appropriate depth of the mucus layer.

Mucus is simultaneously a protective barrier, a dynamic clearance system, and a potential anchoring site for particulate formulations. Its viscoelastic mucin network can retain particles through electrostatic, hydrophobic, and hydrogen-bonding interactions, while mucus renewal can remove strongly trapped carriers before they reach bacteria or epithelial cells [24,25]. Particle size, surface charge, hydrophilicity, and coating density influence this balance. Two broad design strategies have therefore emerged: mucoadhesion, which increases particle interactions with mucus and can prolong local residence, and mucopenetration, which minimizes adhesive interactions to facilitate diffusion toward the epithelial surface [25,26,27]. Dense hydrophilic coatings, particularly those based on polyethylene glycol (PEG), can improve diffusion through mucus, whereas cationic polymers tend to interact more strongly with negatively charged mucins [26,27]. These approaches are complementary rather than interchangeable, and their value depends on the location of the intended therapeutic target.

The apparent trade-off between mucoadhesion and mucus penetration can be resolved only in relation to the location of the therapeutic target. Strong adhesion may prolong local residence and drug exposure within accessible mucus, but it can also immobilize a carrier in superficial mucin layers, where it may be cleared during mucus turnover before reaching *H. pylori* closer to the epithelium. In contrast, densely hydrated, minimally adhesive surfaces can facilitate transport to deeper sites but may provide less adhesive retention. Accordingly, mucin binding, mucus residence, and transport through mucus should be measured as distinct endpoints. Changes in nanoparticle zeta potential after incubation with mucin indicate interactions with mucin but do not establish diffusion through an intact mucus layer; permeability experiments, particle tracking, or spatial imaging are needed for that conclusion [24,25,26,27].

Chitosan illustrates this design trade-off particularly well. Protonation of its amino groups under acidic conditions promotes interaction with mucin glycoproteins and can enhance gastric retention [28,29,30,31], but excessive adhesion may restrict deeper transport. Its chemistry can also be modified to alter pH stability and biological interactions. In studies by Gonciarz et al., spray-dried chitosan microparticles carrying viable *Mycobacterium bovis* Bacillus Calmette–Guérin (BCG) were prepared either unmodified or modified with N-acetyl-D-glucosamine (GlcNAc) or Pluronic F127. The formulations maintained BCG viability after spray drying and showed formulation-dependent release *in vitro,* while subsequent animal studies examined compartment-dependent delivery *in vivo* [8,9,10].

The original studies illustrate why carrier characterization should be linked to the transport endpoint being assessed. Amoxicillin-loaded cysteine-conjugated chitosan/poly(malic acid) nanoparticles had a mean diameter of 223.7 ± 3.2 nm and a zeta potential of +19.4 ± 0.2 mV; they interacted with mucin and pH-dependent drug release, but the reported assays did not directly establish transport through a complete gastric mucus layer [30]. Amoxicillin-loaded chitosan nanoparticles co-administered with inulin measured 157.8 ± 3.85 nm and exhibited sustained release over 72 h, whereas their antibacterial and *Lactobacillus casei* viability measurements likewise did not constitute direct measurements of mucus penetration [31]. Conversely, the PEG-functionalized lipid–polymer platform of Li et al. was tested for passage through mucus and disruption of an underlying *H. pylori* biofilm, thus providing a more direct link between surface engineering and barrier traversal [17]. The BCG-loaded chitosan formulations should be assessed separately as 1–20 μm spray-dried microparticles that released viable bacilli in a formulation-dependent manner under acidic or near-neutral to alkaline conditions, not as mucus-penetrating nanoparticles [8].

In precancerous gastric mucosa, epithelial access is a distinct objective that extends beyond luminal retention. Gastric epithelial cell membrane-coated nanomedicines developed by Ma et al. were examined for stability under simulated gastric acidity, transport in porcine gastric mucus, epithelial localization, and hydrogen peroxide-responsive destabilization. Reported hydrodynamic diameters included 155.0 nm for the GES-1 membrane-derived formulation and 167.2 nm with a zeta potential of −34.8 mV for the corresponding murine epithelial membrane-based preparation [19]. These endpoints provide preclinical evidence for distinct delivery steps, but neither particle size measurements nor improvements in metaplasia-associated endpoints establish long-term prevention of gastric cancer.

### 2.2. Systemic Tumor-Directed Delivery: Circulation, Extravasation, and Tissue Penetration

A dense extracellular matrix (ECM), elevated interstitial pressure, heterogeneous vascularization, hypoxia, local acidosis, and spatially distinct proliferative states can limit particle distribution and produce uneven drug exposure within tumor tissue [13,32]. Gastric nanomedicine therefore requires stage- and target-adapted design: prolonged residence can be useful for local anti-*H. pylori* therapy, carefully controlled mucus interactions are needed to reach bacteria or the gastric epithelium, and anticancer nanocarriers must additionally penetrate a structurally and molecularly heterogeneous tumor microenvironment.

Orally or locally administered anti-infective systems and intravenously delivered anticancer nanocarriers therefore confront fundamentally different sequences of barriers. The former must maintain function in gastric fluids, negotiate mucus renewal, and reach bacterial or epithelial sites. The latter must first remain stable in blood, limit clearance associated with protein-corona formation and uptake by the mononuclear phagocyte system, extravasate through heterogeneous tumor vessels, and then move through interstitial and extracellular-matrix barriers before intracellular delivery [13,32,33]. For example, folate-modified, pH/ROS-responsive docetaxel nanoparticles had a reported diameter of 220.0 ± 5.1 nm and zeta potential of approximately −30 mV; fluorescently labeled formulations were tracked within HGC-27 spheroids and in mouse xenografts after intravenous administration [34]. By contrast, the distribution of doxorubicin-associated fluorescence in AGS spheroids treated with PLA-His-DOX does not, on its own, establish that intact polymer nanoparticles reached the spheroid interior [22,35]. Table 1 compares representative physicochemical characteristics with the actual delivery evidence and limitations of the respective models.

## 3. Nanomaterials for the Control of *H. pylori* Infection

The increasing difficulty of *H. pylori* eradication has encouraged nanomaterial-based strategies that address several weaknesses of conventional therapy at once. Nanocarriers can protect labile drugs, increase local residence, modify release kinetics, and improve access to bacteria located beneath the mucus layer. Polymeric systems are especially attractive because their composition and surface properties are readily tunable. Thiolated chitosan/poly(malic acid) nanoparticles (NPs), for example, were developed as pH-sensitive carriers for amoxicillin and showed mucoadhesive characteristics together with pH-dependent drug release [30]. In another approach, amoxicillin-loaded chitosan NPs were co-administered with prebiotic inulin. The encapsulated antibiotic exhibited sustained release and enhanced antibacterial activity, while inulin helped preserve the viability of *L. casei* under the tested conditions [31]. Such studies show a shift from simple antibiotic encapsulation toward delivery systems designed around local efficacy and microbiota compatibility. These experimental approaches should currently be regarded as complementary approaches rather than replacements for guideline-based *H. pylori* eradication regimens [6].

Lipid-based carriers provide a distinct alternative to polysaccharide nanoparticles. Lopes-de-Campos et al. encapsulated amoxicillin in solid lipid nanoparticles containing cetyl palmitate, Tween 80, linolenic acid, and dioleoylphosphatidylethanolamine; *in vitro* tests supported protection under acidic conditions, interactions with mucin, and epithelial compatibility, but did not establish eradication efficacy in infected animals [36]. In another experimental study, Wu et al. incorporated mannosylerythritol lipid-B into phospholipid/cholesterol nanoliposomes. Amoxicillin-loaded vesicles (~100 nm) showed improved stability under simulated gastric conditions, mucus and biofilm penetration *in vitro,* and antibacterial effects in infected mice [37]. Thus, lipid composition can affect gastric stability and bacterial access, although the strength of evidence for delivery and therapeutic efficacy varies between studies.

A major challenge is that an effective anti-*H. pylori* system may need to cross both gastric mucus and the extracellular polymeric matrix of a bacterial biofilm. Li et al. addressed these sequential barriers using lipid–polymer NPs with a chitosan core, a rhamnolipid-containing lipid shell, polyethylene glycol (PEG) surface functionalization, and encapsulated clarithromycin [17]. PEGylation facilitated mucus penetration, rhamnolipid disrupted the biofilm matrix, and chitosan and clarithromycin contributed to antibacterial activity. Rhamnolipid-loaded chitosan/poly(malic acid) NPs also strongly inhibited *H. pylori* biofilm formation [38], while self-assembled nanodrugs composed of berberine derivatives and rhamnolipids combined mucus penetration, extracellular polymeric substance disruption, inhibition of bacterial re-adhesion, and reduced bacterial burden and inflammatory injury in infected mice [39]. These findings support biofilm disruption as a design function rather than merely an additional microbiological endpoint.

Surface functionalization can further refine interactions with the pathogen. Mannose-modified chitosan/poly(malic acid) NPs showed antibacterial and antibiofilm activity against multidrug-resistant *H. pylori*, while molecular modeling examined binding to an *H. pylori* lectin implicated in bacterial adhesion [40]. This type of approach represents a promising strategy because disrupting adhesion may complement direct bacterial killing and limit recolonization.

Nanomaterials may also exert intrinsic antibacterial activity without serving primarily as antibiotic carriers. Metal- and metal-oxide-based systems may disrupt bacterial membranes, release antimicrobial ions, generate reactive oxygen species (ROS), or exert catalytic and photothermal effects [41]. Silver NPs are among the most widely studied examples. In infected gastric epithelial AGS cells, silver NPs inhibited *H. pylori* growth and modified CagA-associated cellular responses, demonstrating that nanoscale materials can influence both bacterial viability and virulence-linked host signaling [42]. More sophisticated platforms attempt to couple bactericidal action with control of the inflammatory consequences of infection. A cerium oxide nanozyme responsive to gastrointestinal conditions generated bactericidal superoxide radicals under gastric conditions but scavenged excess ROS in the epithelial environment, reducing inflammation while causing limited disruption of the commensal microbiota [43].

Inorganic and catalytic platforms should be distinguished from carriers that merely encapsulate antibiotics. Silver nanoparticles were evaluated for antibacterial activity and effects on CagA-associated host–cell responses [42]. The cerium oxide-based CeOSL nanozyme provides a stimulus-responsive example: its oxidase-like activity was activated in gastric juice and mucus, whereas antioxidant activity under epithelial conditions was associated with reduced oxidative injury [43]. These are preclinical effects, and the local generation of bactericidal reactive oxygen species must be balanced against possible host toxicity.

Antibiotic-free multifunctional systems extend this approach by addressing extracellular bacteria, biofilms, intracellular reservoirs, and mucosal repair within a single therapeutic platform. A chitosan-coated lipid/copper-organic framework platform developed by Lai et al. combined rhamnolipid-mediated biofilm dispersion, phosphatidic-acid-driven lysosomal acidification and autophagy, and controlled Cu^2+^ release [18]. The system eliminated *H. pylori* reservoirs, attenuated inflammation, promoted mucosal repair, and was designed to minimize disruption of the intestinal microbiota. Such pathogen-oriented multifunctionality is especially relevant to *H. pylori*, where treatment failure can result from several spatially distinct bacterial niches rather than antibiotic susceptibility alone.

Hybrid platforms combine material classes to address successive barriers rather than simply increasing the number of payloads. In the lipid–polymer nanoparticles developed by Li et al., a chitosan core carried clarithromycin, a rhamnolipid-containing lipid layer facilitated biofilm disruption, and PEG modification facilitated mucus transport [17]. The lipid/copper-organic-framework system of Lai et al. instead combined a chitosan coating, biofilm-disrupting components, intracellular-reservoir targeting, and controlled copper release [18]. These examples illustrate experimentally investigated hybrid architectures, while their clinical utility remains to be established.

A complementary route is host-directed immunomodulation, which remains experimental and is not an established eradication strategy [8,9,10,12]. Gonciarz et al. incorporated viable *M. bovis* BCG into spray-dried chitosan microparticles modified with GlcNAc or Pluronic F127 [8]. In subsequent *Cavia porcellus* studies [9,10], oral administration enabled formulation-dependent release of viable BCG in the stomach or intestine and was associated with reduced gastric *H. pylori* colonization, reduced levels of mucin associated with bacterial attachment, together with T-lymphocyte infiltration patterns and cytokine profiles consistent with an immunoregulatory rather than pro-inflammatory function [9,10]. This work broadens the therapeutic concept from direct bacterial killing toward modulation of host–microbe interactions and immune responses.

## 4. From *H. pylori* Infection to a Pro-Tumor Gastric Microenvironment: Potential Interventions

Persistent *H. pylori* infection promotes gastric carcinogenesis not only through direct bacterial effects on epithelial cells but also by progressively reshaping the surrounding immune, stromal, and extracellular compartments. This chronic inflammatory niche provides a mechanistic link between colonization and malignant transformation. CagA, vacuolating cytotoxin A (VacA), bacterial surface components, and recurrent epithelial injury activate interconnected pathways that include NF-κB, signal transducer and activator of transcription 3 (STAT3), MAPK, Wnt/β-catenin, and Hippo/YAP signaling, thereby influencing cytokine production, cell survival, proliferation, polarity, and differentiation [2,3]. The resulting microenvironment is dynamic: epithelial damage and regeneration occur alongside immune-cell recruitment and stromal activation.

Oxidative and nitrosative stress are central components of this process. Activated neutrophils, macrophages, and epithelial cells generate ROS and reactive nitrogen species (RNS), which can oxidize lipids and proteins, induce DNA lesions and strand breaks, and contribute to genomic instability [3]. Repeated tissue injury also creates conditions in which proliferating epithelial cells are continuously exposed to inflammatory and genotoxic signals. In a study by Gonciarz et al. using primary gastric epithelial cells and fibroblasts, *H. pylori* lipopolysaccharide (LPS) increased oxidative stress and apoptosis and impaired interleukin-33 (IL-33)-driven cell migration and wound repair, with accompanying changes in collagen I production [44]. These findings suggest that bacterial components can disturb physiological regeneration as well as directly injure the epithelial barrier.

The infection-associated niche also involves substantial stromal and immune reprogramming. *H. pylori* alters macrophage behavior, regulatory T-cell responses, and other immune cell populations in ways that can sustain inflammation while progressively weakening effective antitumor surveillance [2,3]. In parallel, inflammatory signaling modifies the ECM and activates fibroblasts. One mechanistic example is the NF-κB–PIEZO1–YAP1–CTGF axis, through which *H. pylori*-driven signaling promoted cancer-associated fibroblast (CAF) accumulation and collagen deposition; the resulting tissue stiffening formed a positive feedback loop that supported GC progression and reduced responsiveness to chemotherapy [45]. Thus, infection can influence not only malignant epithelial cells but also the physical and cellular properties that determine therapeutic access and response.

Recent single-cell analyses further indicate that aspects of this remodeling may persist after bacterial eradication. Fu et al. identified immunomodulatory CAF populations associated with ECM reorganization, epithelial stemness, and immunosuppression in *H. pylori*-associated disease. Selected CAF states remained enriched in advanced precancerous mucosa after eradication, particularly in cases with limited histopathological improvement [14]. This experimental observation is consistent with clinical evidence that eradication reduces but does not eliminate subsequent GC risk. A 2025 systematic review and meta-analysis identified severe gastric atrophy and intestinal metaplasia as major risk factors for GC after eradication, supporting continued surveillance of patients with advanced mucosal damage [15]. Together, these findings suggest that removal of the bacterium may not fully reverse an established pro-neoplastic tissue program.

**Figure 1:**
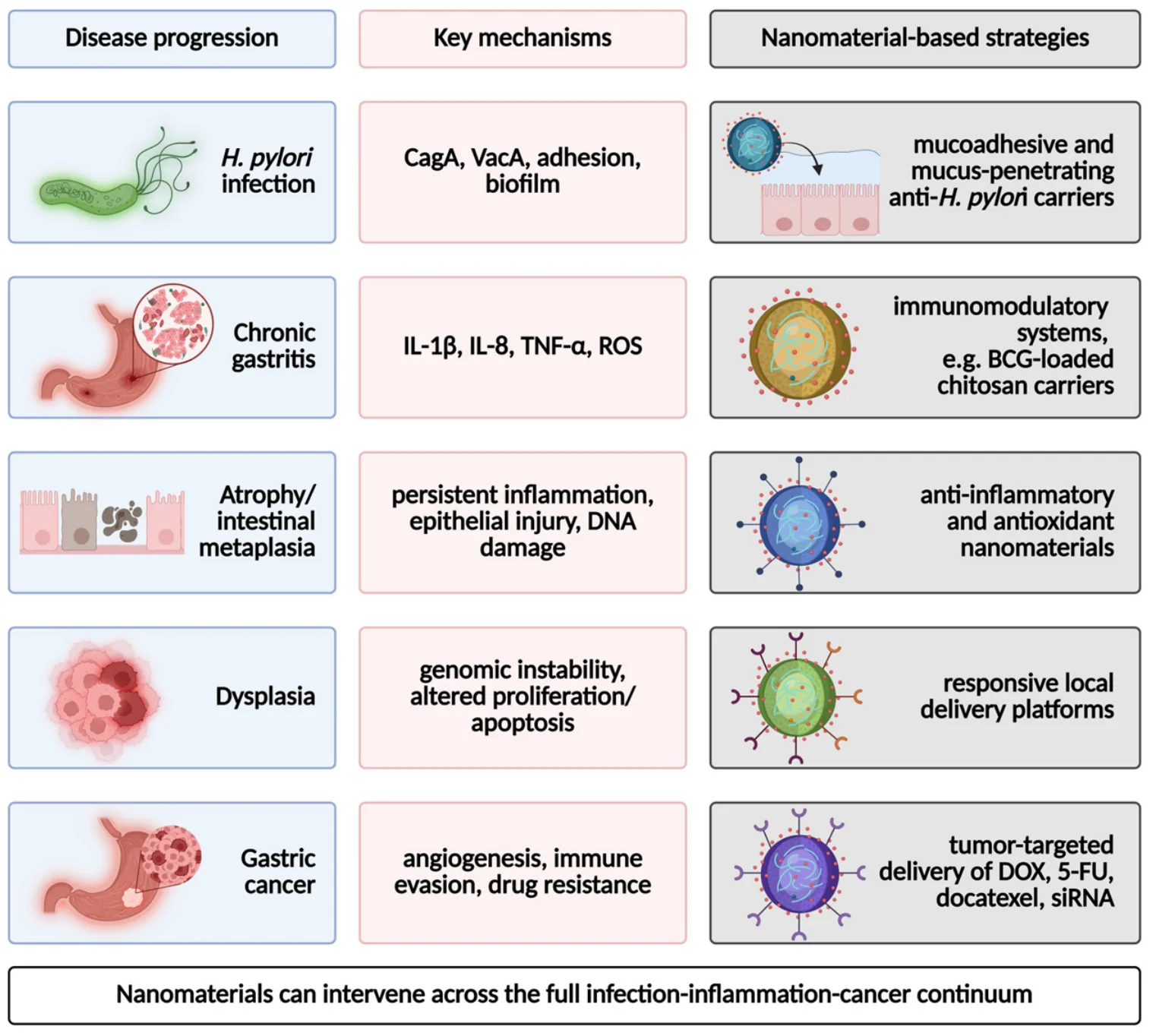
Overview of experimental nanomaterial-based interventions along the *H. pylori*–inflammation–gastric cancer continuum. Abbreviations: *H. pylori*, *Helicobacter pylori*; *GC*, gastric cancer; ROS, reactive oxygen species; ECM, extracellular matrix; NPs, nanoparticles; BCG, Bacillus Calmette–Guérin. Created in BioRender. Mikolajczyk-Chmiela, M. (2026) https://BioRender.com/dredgoz.

An infection-conditioned environment may also modify therapeutic responses. In the AGS spheroid model developed by Jaroniek et al., CagA or LPS was included when evaluating histamine-functionalized poly(lactic acid) (PLA) nanoparticles loaded with doxorubicin (PLA-His-DOX) [22]. In selected comparisons, IL-1β and/or IL-8 levels were lower after treatment with PLA-His-DOX together with bacterial components than after exposure to the respective bacterial components alone; however, those components did not significantly increase cytokine concentrations relative to unstimulated controls, and the concentrations were not normalized to the number of viable cells [22]. These findings support evaluating therapeutic responses in the presence of defined bacterial cues, but do not establish an induced inflammatory microenvironment or a distinct anti-inflammatory effect of PLA-His-DOX [22]. Collectively, oxidative stress, altered epithelial repair, CAF activation, ECM remodeling, and immune dysregulation define a pro-tumor environment relevant to carcinogenesis and therapeutic design. The progression from infection-associated mucosal injury to a pro-tumor gastric microenvironment, together with potential nanotherapeutic intervention points, is summarized in Figure 1.

## 5. Nanomaterials for Gastric Cancer Therapy

Nanomaterial-based drug delivery has been extensively explored in GC because it can improve the solubility and stability of therapeutic cargo, modulate release kinetics, and increase drug exposure in tumor tissue while potentially reducing nonspecific systemic toxicity [13,32]. These advantages are relevant in advanced GC, where molecular heterogeneity, incomplete tumor penetration, immunosuppressive signaling, and acquired drug resistance can limit treatment efficacy. Current approaches include polymeric and lipid nanocarriers, albumin-based formulations, inorganic and hybrid materials, and multifunctional platforms combining molecular targeting with stimuli-responsive release, gene regulation, phototherapy, or immune modulation.

For intravenously administered formulations, favorable pharmacokinetics and transport from the bloodstream are prerequisites for tumor accumulation; particle penetration into gastric mucus is generally not the main barrier. In contrast, a locally administered formulation intended to act at the gastric epithelial surface must still address luminal stability and mucus transport. Therefore, similar nominal particle sizes do not imply comparable delivery performance across these distinct routes, and cellular uptake, spheroid distribution, and tumor accumulation should be reported as separate endpoints (Table 1) [13,32,33,34].

Importantly, most tumor-directed nanocarriers described in this section have been assessed for anticancer efficacy rather than for effects on *H. pylori*-dependent mechanisms. Unless bacterial infection, defined *H. pylori* components, or infection-associated tissue remodeling are part of the experimental model, efficacy against gastric cancer cells should not be interpreted as evidence of bacterial eradication, reversal of premalignant lesions, or interruption of infection-driven carcinogenesis.

Polymeric nanoparticles based on PLA, poly(lactic-co-glycolic acid) (PLGA), or chitosan can carry cytotoxic drugs, natural compounds, nucleic acids, and molecular inhibitors, while their degradation rate, surface charge, and ligand density can be tailored to a specific biological objective [13,32]. Liposomes, solid lipid NPs, nanostructured lipid carriers, micelles, and albumin-based systems provide complementary options, particularly for poorly soluble drugs. In all cases, additional formulation complexity is justified only when it improves exposure at the intended site, rather than merely increasing cellular uptake in simplified *in vitro* models.

The therapeutic role of each material class also differs in GC. Polymeric PLGA and PLA systems are exemplified by co-delivery of docetaxel and LY294002 [21] and histamine-functionalized carriers containing doxorubicin or 5-fluorouracil [35,46,47]. A clinically relevant protein-based comparator is albumin-bound paclitaxel, which should not be confused with evidence for infection-directed therapy [48]. Hybrid tumor-directed platforms include gastric-cancer-cell-membrane-coated PLGA particles delivering STM2457 [49], while pH/ROS-responsive docetaxel nanoparticles exploit tumor-associated microenvironmental triggers [34]. Although inorganic carriers are frequently proposed for catalytic, imaging, or photothermal applications [13,32], the present review does not imply that all such applications have been independently validated for *H. pylori*-associated GC.

A central design question is whether tumor accumulation is expected to arise from passive or active targeting. The enhanced permeability and retention (EPR) effect can facilitate accumulation of nanoscale carriers in some tumors, but its magnitude is heterogeneous and cannot be assumed to be uniform across GC lesions [50]. Active targeting uses antibodies, peptides, or other ligands to recognize tumor-associated surface molecules and enhance cellular interaction after the carrier reaches the tumor [13,32]. Human epidermal growth factor receptor 2 (HER2)-targeted NPs carrying the maytansinoid derivative DM1 showed preferential activity in HER2-positive NCI-N87 cells and increased tumor accumulation *in vivo* [20]. Gastric-cancer-cell-membrane-coated PLGA NPs functionalized with an ephrin type-A receptor 2 (EphA2)-binding YSA peptide have also been used to deliver the methyltransferase-like 3 (METTL3) inhibitor STM2457, suppress MYC proto-oncogene protein (c-MYC) and bromodomain-containing protein 4 (BRD4), reduce programmed death-ligand 1 (PD-L1), and enhance responses to anti-programmed cell death protein 1 (PD-1) treatment in preclinical models [49].

Stimuli-responsive systems add a further level of control by exploiting acidity, hypoxia, elevated ROS, enzyme activity, or redox imbalance [13,32]. Hypoxia is particularly relevant because hypoxia-inducible factor 1 alpha (HIF-1α) promotes angiogenesis, epithelial–mesenchymal transition, metabolic adaptation, and resistance-related programs in GC [51]. Responsive carriers can therefore be designed either to release cargo under hypoxic conditions or to deliver agents that interfere with hypoxia-associated signaling. Inorganic and hybrid materials additionally provide photothermal, photodynamic, magnetic, catalytic, imaging, and, for high-atomic-number materials, radiosensitizing functions.

Overcoming multidrug resistance (MDR) is another important application. Nanoparticle internalization by endocytosis can partially bypass membrane-level drug-efflux mechanisms mediated by ATP-binding cassette transporters, including P-glycoprotein, while intracellular release can increase drug exposure despite active efflux [52]. Nanocarriers can also co-deliver cytotoxic agents with small interfering RNA (siRNA) or inhibitors directed against resistance pathways such as B-cell lymphoma 2 (Bcl-2), NF-κB, PI3K/Akt, or HIF-1α [13,32,52]. Cai et al. developed PLGA NPs co-encapsulating docetaxel and the PI3K inhibitor LY294002; the formulation showed stronger pro-apoptotic and antitumor effects in cell-based and mouse GC models than the corresponding single-agent formulations [21]. These approaches are attractive because they target the biological mechanism of resistance rather than simply increasing the administered dose. In *H. pylori*-associated GC, these resistance mechanisms may additionally operate within an inflammation- and hypoxia-conditioned tumor microenvironment, further supporting context-adapted nanocarrier design.

The biological effects of GC nanotherapy extend beyond conventional cytotoxicity. Depending on composition and cargo, nanomaterials can induce mitochondrial dysfunction, oxidative stress, DNA damage, cell-cycle arrest, apoptosis, ferroptosis, or reductions in proliferation and migration [13,35,46,53]. Multifunctional systems can also reprogram the tumor immune microenvironment; a PLGA platform conjugated with an internalizing Arg-Gly-Asp (iRGD) peptide co-delivering small interfering RNA against SRY-box transcription factor 9 (siSOX9), chlorin e6, and L-arginine promoted dendritic-cell maturation and CD8^+^ T-lymphocyte activation while suppressing tumor growth in preclinical GC models [54].

Most targeted, responsive, MDR-oriented, and immunomodulatory examples discussed above remain preclinical. Clinical validation remains limited. Albumin-based carriers are attractive because albumin is biocompatible, can bind hydrophobic molecules, and allows selected drugs to be formulated without conventional solvents. Nanoparticle albumin-bound paclitaxel (nab-paclitaxel) provides an important clinical benchmark: in the phase III ABSOLUTE trial, weekly nab-paclitaxel was non-inferior to weekly solvent-based paclitaxel with respect to overall survival in patients with previously treated advanced GC [48]. Histamine-functionalized PLA nanoparticles initially reported by Brzeziński et al. and subsequently evaluated by Jaroniek et al. provide a complementary experimental example of how polymer end-group chemistry can alter cell–carrier interactions and therapeutic responses [35,46,47]; their mechanistic effects are discussed in the following section.

## 6. Functionalized Polymeric Nanocarriers: Histamine-Terminated PLA as a Case Study

Surface and end-group functionalization can alter nanoparticle charge, interfacial interactions, drug release, and intracellular processing, making polymer chemistry a determinant of biological activity rather than merely a structural feature. PLA is a useful model because it is biodegradable and can be synthesized with defined terminal groups. Brzeziński et al. introduced histamine (His) as a PLA terminal group to produce PLA-His NPs [47]. In this context, His should not be regarded as a validated receptor-specific targeting ligand; rather, the imidazole-containing end group modifies carrier properties and cell–particle interactions.

The initial proof-of-concept used doxorubicin (DOX)-loaded PLA-His nanoparticles (PLA-His-DOX). Compared with hydroxyl-terminated PLA formulations, the His-functionalized carriers showed greater activity against AGS gastric adenocarcinoma cells under the tested conditions [47]. Subsequent mechanistic work demonstrated increased oxidative damage and DNA damage, enhanced apoptosis, and reduced proliferation [35]. Changes in the tight-junction protein zonula occludens-1 (ZO-1) and increased expression of intercellular adhesion molecule 1 (ICAM-1) further indicated that surface or end-group chemistry can influence epithelial integrity and adhesion-molecule expression, although these findings do not establish receptor-mediated targeting or an in vivo immune benefit.

The carrier concept was subsequently extended to 5-fluorouracil (5-FU). PLA-His-5-FU reduced mitochondrial membrane potential, promoted apoptosis and cell-cycle arrest, and inhibited AGS-cell proliferation [46]. In the reported *in vitro* experiments, the formulation increased TNF-α and IL-1β secretion in AGS cells and activated NF-κB-associated signaling in THP-1 Blue monocytes [46]. Such changes should be interpreted as context-dependent biological responses rather than evidence of a consistently beneficial immunomodulatory effect. Together, these studies illustrate of how polymer end-group chemistry can alter the response to an encapsulated anticancer drug.

## 7. Advantages of Infection-Associated 3D Models of Gastric Cancer

Conventional two-dimensional monolayers remain valuable for mechanistic screening, but they capture only a limited proportion of the structural constraints encountered by nanomaterials in solid tumors. Cells on plastic experience relatively uniform access to oxygen, nutrients, and therapeutics, whereas tumors contain spatial gradients, heterogeneous cell states, ECM barriers, and regions with markedly different proliferative activity. Three-dimensional GC models, particularly multicellular tumor spheroids and patient-derived organoids, better reproduce several of these features and can provide more demanding tests of drug and nanoparticle penetration [55].

Spheroids are especially useful for nanomedicine because their architecture creates diffusion gradients for oxygen, metabolites, and therapeutic compounds. Proliferating cells are generally enriched toward the periphery, whereas deeper regions contain quiescent or necrotic cell populations as spheroids increase in size. A formulation that appears highly active in a monolayer may therefore show weaker effects when transport through the multicellular structure limits drug exposure [55]. Organoids add further biological complexity by preserving tissue architecture and, in patient-derived systems, aspects of interindividual heterogeneity. Gastric organoids are also established tools for studying *H. pylori*–epithelial interactions, inflammation, and early disease mechanisms [56].

The design of a penetration experiment should distinguish the movement of an intact nanocarrier from the distribution of fluorescent cargo released from the carrier. Confocal optical sections or light-sheet imaging, size-dependent tracer controls, and quantitative radial fluorescence profiles can characterize spatial drug or particle distribution; dual labeling of the carrier and drug enables a more direct assessment of their dissociation. In parallel, spheroid diameter, viability, apoptosis, and drug-response measurements are needed because cytotoxicity may alter tissue architecture and the apparent extent of penetration. These readouts are not interchangeable, and multicellular organization does not by itself recreate gastric mucus, bacteria, or an immune microenvironment [22,34,55].

For *H. pylori*-associated GC, however, three-dimensional architecture alone is not sufficient. Here, the term infection-associated model refers to a three-dimensional system that incorporates defined microbial or inflammatory cues; it does not necessarily imply live bacterial infection. Depending on the scientific question, these cues may include controlled exposure to live bacteria, purified bacterial components, or defined cytokine conditions. Such models can test whether infection-associated signaling modifies nanoparticle uptake, penetration, cytotoxicity, inflammatory mediators, or sensitivity to chemotherapy while allowing individual experimental variables to be assessed separately.

These approaches differ in what they can demonstrate. Exposing epithelial or tumor-only spheroids to purified CagA or LPS enables selected responses to bacterial components to be studied, but cannot reproduce bacterial adhesion, persistent colonization, or type IV secretion system-dependent delivery of CagA. Models incorporating live *H. pylori* can examine host–pathogen interactions, but infection of epithelial cells alone does not reproduce the full immune and stromal context. Multicellular co-cultures that incorporate macrophages or other immune cells, optionally alongside fibroblasts, enable interactions between host–cell populations to be investigated, although their relevance depends on which components of the gastric microenvironment they actually represent [22,55,56].

Gastric epithelial organoids offer a complementary infection model: Bartfeld and Clevers described cultures derived from human and mouse gastric glands in which viable *H. pylori* was introduced into the organoid lumen by microinjection [57]. Such models are informative for bacterial–epithelial interactions, but the cited protocol was not a nanoparticle-penetration study; the limited throughput of microinjection, organoid orientation, and lack of immune and stromal cells limit direct extrapolation. For cancer-focused drug screening, Xu et al. generated gastric cancer patient-derived organoids retaining selected histological and molecular features of their tumors and compared sensitivity to conventional and targeted agents [58]. These results support personalized drug-response testing, but do not independently validate nanoparticle penetration or the simulation of active infection.

Jaroniek et al. used AGS spheroids to examine responses to defined *H. pylori* components together with the effects of PLA-His-DOX [22]. Spheroids were exposed to CagA or bacterial LPS and then assessed for growth and treatment-related effects. PLA-His-DOX reduced MTT-based metabolic activity and induced DNA-damage and apoptosis-related responses. The cytokine results require a more cautious interpretation: *H. pylori* components did not significantly increase IL-1β or IL-8 versus unstimulated controls in the main comparisons, although selected comparisons showed lower cytokine concentrations when bacterial components were combined with DOX or PLA-His-DOX than when the corresponding bacterial component was used alone [22]. IL-1β and IL-8 were quantified by ELISA in culture supernatants after 24 and 72 h, without normalization to viable cell number, DNA content, or total cellular protein [22]. Because PLA-His-DOX reduced MTT-based metabolic activity and was associated with apoptosis-related readouts, lower cytokine concentrations may reflect a reduction in viable cell number, altered secretion per surviving cell, or both. These possibilities cannot be separated using the reported data, so the results do not independently demonstrate an anti-inflammatory mechanism or a consistent cytokine-defined inflammatory phenotype. Accordingly, this model is most accurately described as an AGS tumor spheroid model exposed to defined *H. pylori* components suitable for studying tumor-cell responses to defined bacterial cues, rather than a model of active or chronic infection.

To assess whether a treatment directly alters inflammatory signaling, cytokine output should be interpreted alongside contemporaneous measures of viable cell number (or another independently validated normalization measure) and additional inflammatory readouts. Comparisons with appropriately controlled live-infection systems and immune-cell-containing co-cultures could clarify the contributions of viable bacteria and multicellular interactions to the observed responses. These analyses remain future validation steps rather than results of the cited AGS spheroid study [22].

A practical approach is therefore to select each model according to the endpoints it can reliably measure: bacterial attachment and infection responses in microinjected gastric organoids; drug and fluorescence distribution in tumor spheroids; and interpatient variability in treatment responses in tumor organoids. Co-culture with immune cells or fibroblasts can add relevant biological complexity, but any claim of predictive superiority requires model-specific validation. Table 2 summarizes cell types, characteristic features, direct evidence, and the limitations of representative models.

## 8. Next-Generation Dual-Action Nanotherapeutics

The next generation of nanotherapeutics for *H. pylori*-associated GC should move beyond the assumption that “multifunctional” necessarily means loading multiple agents into an increasingly complex single particle. A more useful concept of dual action involves integrating two biologically related therapeutic functions, such as bacterial clearance with inflammation control, pathogen clearance with restoration of the premalignant mucosa, or tumor-cell killing with modulation of a treatment-limiting microenvironment. The appropriate combination should be determined by disease stage because infection, chronic inflammation, precancerous remodeling, and established malignancy are linked but do not necessarily coexist. Thus, stage-adapted modularity does not imply that BCG, antibacterial agents, and anticancer drugs have been successfully co-encapsulated or should necessarily be combined within a single particulate formulation.

Several systems already illustrate this principle. The gastrointestinal-responsive cerium oxide nanozyme combines bactericidal ROS generation under gastric conditions with ROS scavenging and anti-inflammatory effects at the epithelial interface [43]. An antibiotic-free chitosan-coated copper-organic framework platform integrates elimination of planktonic, biofilm-associated, and intracellular *H. pylori* with autophagy activation, inflammation reduction, mucosal repair, and microbiota preservation [18]. In both examples, the additional function addresses a biological consequence or bacterial reservoir that can undermine eradication.

A broader strategy is to link infection control with intervention at the premalignant stage. orally administered nanomedicines designed to localize to gastric epithelial cells developed by Ma et al. combined resveratrol, lactoferrin, and modified fucoidan within a biomimetic membrane-coated platform designed for acid resistance, mucus penetration, epithelial localization, and H_2_O_2_-responsive release [19]. The system promoted intracellular *H. pylori* clearance through sirtuin 1 (SIRT1)-associated autophagy and improved features of intestinal metaplasia, linking pathogen removal with restoration of mucosal homeostasis. Direct integration of antibacterial and anticancer activity is less developed. Curcumin-stabilized silver NPs showed anti-H. pylori activity, including urease inhibition, and antiproliferative effects in AGS cells [59], but the safety and simultaneous *in vivo* efficacy of such dual-purpose systems remain to be established. These results were obtained in preclinical models and do not demonstrate prevention of gastric cancer, reduced cancer recurrence, or improved survival in patients.

The choice of material may also facilitate modular design. Chitosan is especially attractive because its amino and hydroxyl groups permit chemical functionalization and attachment of antimicrobial, antioxidant, or anticancer components, while its mucoadhesive behavior can support gastric residence [13]. However, this chemical versatility does not establish dual therapeutic efficacy; each added function requires independent biological validation.

For established GC, dual action may instead operate entirely within the tumor compartment. Docetaxel-loaded pH/ROS-responsive NPs improved spheroid penetration and antitumor activity *in vivo* [34], whereas the iRGD-modified PLGA system discussed above combined photodynamic injury with immune reprogramming [54]. The most realistic future strategy may therefore be modular or sequential rather than relying on a universal “all-in-one” formulation. During active infection, priorities are gastric residence, bacterial clearance, biofilm or intracellular-reservoir control, and reduction of inflammatory injury. In persistent atrophy or intestinal metaplasia, restoration of epithelial homeostasis becomes increasingly important, whereas established GC requires tumor penetration, molecular targeting, responsive drug release, and resistance control.

**Figure 2:**
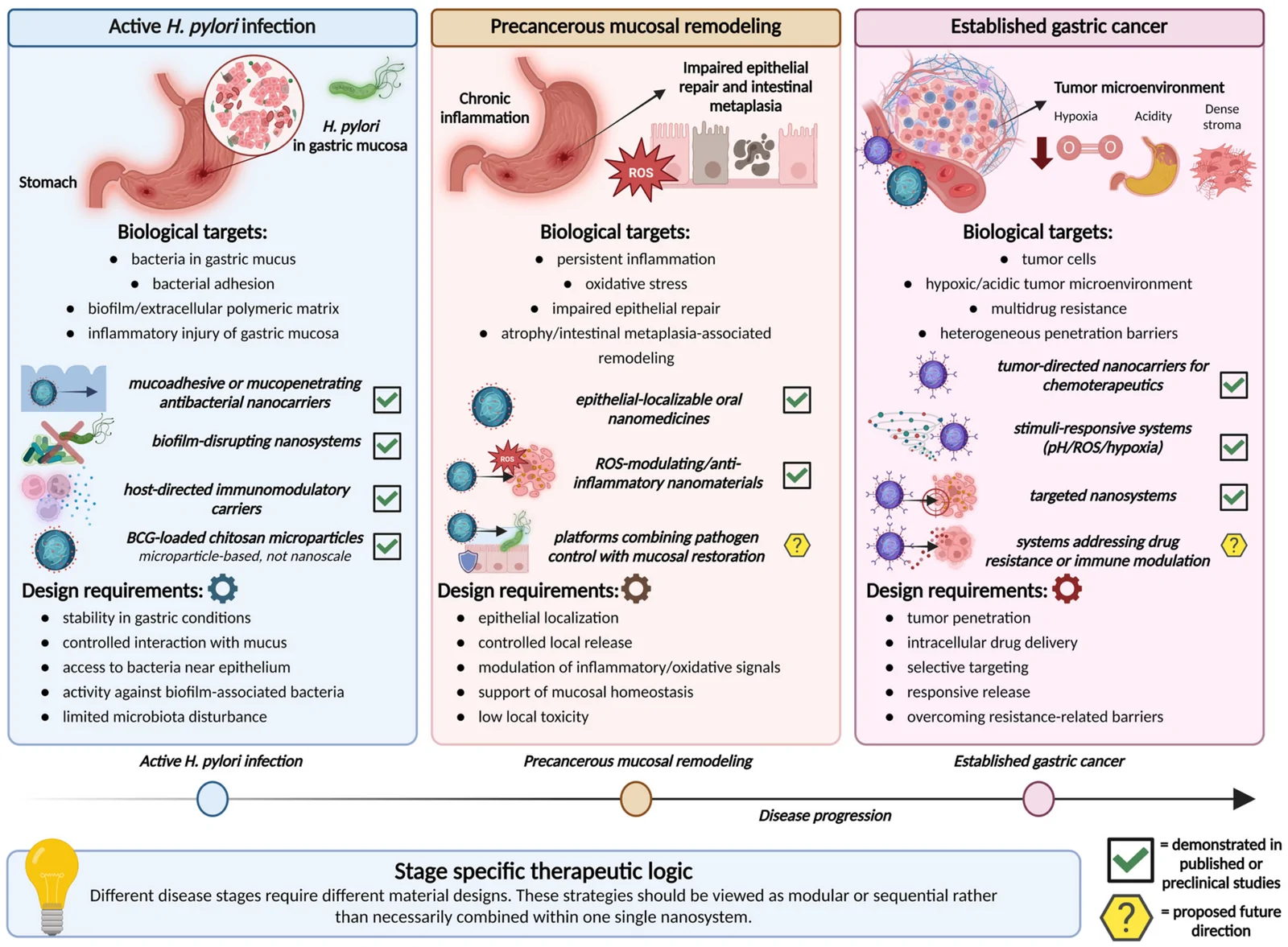
Stage-adapted nanomaterial strategies across the *Helicobacter pylori*–gastric cancer continuum. The panels compare biological targets, representative experimental platforms, and design requirements at the infection, precancerous, and established cancer stages. Solid outlines denote published preclinical examples, whereas dashed outlines indicate proposed future directions. BCG-loaded chitosan carriers are microparticles studied for infection-associated immunomodulation, not constituents of a validated single antimicrobial–anticancer nanosystem. The scheme does not imply proven clinical benefit, reduced recurrence, or improved survival. Created in BioRender. Mikolajczyk-Chmiela, M. (2026) https://BioRender.com/dqr0rel. Abbreviations: BCG, Bacillus Calmette–Guérin; *H. pylori*, *Helicobacter pylori*; ROS, reactive oxygen species.

Studies by Gonciarz et al. and Jaroniek et al. illustrate two distinct experimental approaches along this continuum: chitosan-based BCG delivery for host-directed modulation of *H. pylori* infection [8,9,10] and His-functionalized PLA NPs for delivery of DOX or 5-FU to GC cells [35,46,47]. These systems have not been evaluated as a combined therapy. Rather, they illustrate a practical design principle: future nanomedicine should address the dominant barrier and disease-driving mechanism at each clinical stage, adding complexity only when it provides a clear biological advantage. Representative strategies spanning the *H. pylori*–GC continuum are summarized in Table 3, while the proposed stage-adapted modular framework is illustrated in Figure 2. Importantly, the BCG formulations investigated in the cited studies consisted of spray-dried chitosan microparticles containing viable BCG, not BCG-loaded nanoparticles [8,9,10]. The studies evaluated infection-related and immune outcomes and did not test co-delivery of BCG with anticancer drugs or a unified nanosystem for bacterial eradication and tumor killing. Accordingly, Figure 2 depicts independent stage-specific strategies, using solid outlines for experimentally investigated approaches and dashed outlines for future design concepts.

These examples fall into three evidence categories: (i) infection-directed nanomaterials evaluated using bacterial, biofilm, or mucosal endpoints [17,18,39,43], alongside BCG-loaded chitosan microparticles studied in infection models [8,9,10]; (ii) systems assessing *H. pylori* clearance together with precancerous epithelial changes, as shown by Ma et al. [19]; and (iii) GC-directed nanocarriers without demonstrated effects on the pathogen or infection-related carcinogenesis [20,21,34,35,46,47,49,54]. CagA/LPS-exposed AGS spheroids [22] test defined bacterial cues rather than live infection. Findings from these categories should not be interpreted as equivalent forms of evidence.

## 9. Translational Challenges

Despite rapid progress, most gastric nanomedicine studies remain preclinical. One major challenge is biological variability within the gastric environment. For anti-*H. pylori* systems, performance may vary with gastric pH, mucus properties, bacterial distribution, biofilm burden, and microbiota composition. For GC nanocarriers, heterogeneous vascularization, ECM density, interstitial pressure, hypoxia, and variable expression of molecular targets can produce uneven intratumoral distribution [13,32]. Systemically administered particles may also undergo opsonization, protein-corona formation, and uptake by the mononuclear phagocyte system, thereby reducing the fraction of the administered dose that reaches the tumor [33]. Consequently, high uptake or cytotoxicity in homogeneous cell cultures cannot be assumed to predict therapeutic exposure *in vivo*.

A second challenge is the relevance of experimental models. Conventional AGS monolayers and short-term xenografts do not reproduce the prolonged inflammatory history, mucus barrier, stromal architecture, immune-cell interactions, or microbial context of *H. pylori*-associated GC. Spheroids, organoids, multicellular co-cultures, and suitable *in vivo* models should therefore be incorporated at an early stage of preclinical development, with each model selected for the barrier or mechanism being tested [55,56]. Effects on the gastric and intestinal microbiota should also be examined explicitly for antimicrobial and metal-based materials.

A third challenge is scalable and reproducible manufacturing. Scale-up can alter particle size, dispersity, surface charge, drug loading, and release kinetics, and reproducible production is particularly demanding for multifunctional formulations [60,61]. Translation additionally requires standardized assessment of stability, biodegradation, biodistribution, immunogenicity, off-target accumulation, and chronic toxicity. Regulatory complexity increases when a formulation combines multiple active agents, targeting ligands, biological components, or stimulus-responsive functions. Development should therefore prioritize the minimum set of functions required for a clear clinical advantage, together with scalable manufacturing and disease-relevant validation.

## 10. Conclusions and Future Perspectives

Nanomaterials may provide opportunities to intervene at several points along the *H. pylori*–inflammation–GC continuum, but their value depends on matching carrier function to disease biology. For active infection, the priorities are gastric residence, transport through mucus and biofilms, pathogen control, microbiota preservation, and avoidance of sustained inflammatory injury. During premalignant stages, suppression of persistent oxidative and inflammatory signaling and restoration of epithelial homeostasis may remain relevant even after bacterial eradication. Once GC is established, nanocarriers must additionally overcome tumor heterogeneity, stromal barriers, immunosuppression, and therapeutic resistance.

Future research should therefore prioritize stage-specific and disease-informed design over universal multifunctional particles. Dual-action or sequential platforms are most compelling when each component addresses a mechanistically linked barrier, such as bacterial persistence plus inflammation, or tumor-cell killing plus microenvironmental resistance. Equally important will be validation in infection-associated 3D cell culture systems and *in vivo* models that test penetration, selectivity, efficacy, and safety under realistic conditions. A central translational priority is to move from carrier-centered design toward nanomedicine tailored to disease stage. The preclinical data considered here do not support claims that an untested combination formulation could reduce gastric cancer recurrence or improve patient survival.

## Figures and Tables

**Table 1:** Representative material properties, administration routes, and direct evidence of drug delivery in *H. pylori*-directed and gastric cancer nanomedicine.

Administration Route/Representative Platform	Particle Size and Zeta Potential	Surface Features and Release Triggers	Direct Evidence of Delivery	Interpretation and Limitations
A. Oral/local delivery: infection and precancerous gastric mucosa				
Amoxicillin-loaded thiolated chitosan/PMLA NPs [30]	223.7 ± 3.2 nm; +19.4 ± 0.2 mV (drug-loaded)	Cysteine-modified chitosan; mucoadhesive; release assessed at pH 1.2, 6.0, and 7.0	Mucin interactions inferred from changes in surface charge; pH-dependent drug release	No direct measurement of transit across an intact gastric mucus barrier.
Amoxicillin–chitosan NPs + inulin (co-administered) [31]	157.8 ± 3.85 nm; zeta potential not independently verified	Chitosan ionic-gelation NPs; sustained amoxicillin release over 72 h; inulin given separately	Antibacterial tests and *L. casei* viability and drug-release kinetics	Biological efficacy and sustained release do not demonstrate mucus penetration.
PEGylated lipid–polymer NPs with clarithromycin [17]	Numerical particle size and zeta potential not independently verified	Chitosan core; rhamnolipid-containing lipid shell; DSPE–PEG2000	Transport through mucus and eradication of an underlying *H. pylori* biofilm were evaluated	Direct *in vitro* evidence of barrier transport; clinical efficacy *and in vivo* delivery remain unproven.
CHI-BCG microparticles (not NPs) [8]	1–20 µm (SEM); zeta potential not verified	Spray-dried CHI, CHI–GlcNAc or CHI–Pluronic F127; pH 3.0 vs. 7.2/8.0	Viability of encapsulated BCG and pH-dependent release of viable bacilli	Release of viable BCG and host responses do not establish penetration of intact microparticles through mucus.
Epithelial-localizable oral nanomedicines (GLONs) [19]	155.0 nm (GES-1 membrane); 167.2 nm and −34.8 mV (murine membrane variant)	MUC1-rich epithelial cell membrane; gastric acid resistance; H_2_O_2_-responsive core	Permeability through porcine mucus and particle tracking, epithelial uptake and mouse infection model	Provides preclinical evidence linking barrier transport to epithelial effects; long-term prevention of GC remains untested.
B. Systemic/tumor-oriented delivery: established gastric cancer				
Folate-modified docetaxel-responsive NPs [34]	220.0 ± 5.1 nm; ζ approximately −30 mV	FA–PEG decoration of CA-Oxi-αCD carrier; pH/ROS-sensitive release	Cy5-labeled nanoparticle distribution in HGC-27 spheroids and intravenously treated xenograft models	Cellular and tumor fluorescence supports delivery, but the *in vivo* fate of intact particles remains unresolved.
Histamine-functionalized PLA-His-DOX NPs [22,35]	130–250 nm (DLS range); ζ not verified here	Histamine-terminated PLA; doxorubicin cargo; no validated receptor-specific targeting	Cellular uptake by AGS cells and DOX-associated fluorescence in AGS spheroids	Cargo-derived fluorescence alone does not conclusively demonstrate penetration of intact nanoparticles.

Note: Results from different assays should not be compared directly. “Not independently verified” means that a numerical value could not be confirmed in the accessible source text. Cargo-associated fluorescence does not prove intact-particle penetration. Abbreviations: BCG, Bacillus Calmette–Guérin; CHI, chitosan; DOX, doxorubicin; FA, folic acid; NPs, nanoparticles; PEG, polyethylene glycol; PLA, poly(lactic acid); PMLA, poly(malic acid); ROS, reactive oxygen species.

**Table 2:** Representative three-dimensional and infection-associated gastric models: cell composition, experimental uses, and limitations of the evidence for nanomaterial testing.

Model/Cell Types	Structure and Key Characteristics	Infection Context	Evidence and Potential Readouts for Nanomaterials	Major Limitations
AGS tumor spheroids (human GC cells) [22]	Tumor-cell-only 3D aggregates with peripheral and central regions; controlled exposure to CagA or *H. pylori* LPS.	Purified bacterial components.	PLA-His-DOX: growth, metabolic activity, DNA damage, apoptosis, DOX-associated fluorescence, IL-1β/IL-8.	Fluorescence may reflect released DOX; no immune cells; cytokines not normalized to viable cell number.
HGC-27 GC spheroids [34]	Cancer-cell spheroids with diffusion gradients and distinct central and peripheral regions.	No *H. pylori* exposure.	Fluorescence-labeled pH/ROS-responsive docetaxel formulations: spheroid distribution and treatment response.	Does not reconstruct infection, mucus, or full tumor stroma; penetration *in vitro* does not necessarily predict delivery *in vivo.*
NCI-N87 or other GC-line spheroids [55]	Cell-line aggregates with tumor-associated gradients; size and compaction depend on line and culture conditions.	Only when defined bacterial stimuli are introduced.	Potential platform for viability, radial particle/drug distribution, and molecular-response testing; each assay requires validation.	This is a general model class; no *H. pylori*-associated nanoparticle response has been established here for NCI-N87.
Multicellular GC co-culture spheroids [55]	Cancer cells combined with fibroblasts and/or immune cells in a 3D aggregate.	May be supplemented with infection-associated stimuli.	Proposed assessment of stromal restriction, immune signaling, particle uptake, and drug response.	Results depend on cellular composition; model does not validate a specific *H. pylori*-directed nanotherapy.
Gastric epithelial organoids (human/mouse) [57]	Polarized epithelium derived from primary gastric glands, containing a luminal compartment.	Live *H. pylori* introduced into the lumen by microinjection.	Infection and epithelial response; potential application in local-delivery studies following assay validation.	Not a nanoparticle-delivery study; variable accessibility and limited immune/stromal representation.
Patient-derived gastric tumor organoids [58]	Patient-derived tumor cells retain selected histological and molecular features; multiple GC subtypes.	No live *H. pylori* infection.	Differential sensitivity to chemotherapy and targeted drugs; potential future platform for nanocarrier screening.	Drug-response data do not verify nanoparticle penetration; donor-to-donor variability and the absence of immune and vascular compartments.

Note: GC, gastric cancer; DOX, doxorubicin; CagA, cytotoxin-associated gene A protein; *H. pylori, Helicobacter pylori*; IL, interleukin; LPS, lipopolysaccharide; PLA, poly(lactic acid); ROS, reactive oxygen species; 3D, three-dimensional. The term “potential” denotes an application not demonstrated in the cited study and should not be interpreted as direct evidence of intact-nanoparticle penetration.

**Table 3:** Nanomaterial-Based Strategies across *the H. pylori*–Gastric Cancer Continuum.

Disease Stage/Context	Nanomaterial or Particulate Formulation	Cargo/Active Component	Main Target or Function	Experimental Model	Principal Reported Effect	Ref.
*H. pylori* colonization	Thiolated chitosan/poly(malic acid) NPs	Amoxicillin	pH-responsive, mucoadhesive local delivery	*in vitro*	Controlled release and enhanced anti-*H. pylori* activity	[30]
*H. pylori* colonization and microbiota	Chitosan NPs	Amoxicillin (encapsulated) + inulin (co-administered)	Sustained antibiotic delivery with microbiota support	*H. pylori* and *L. casei*	Reduced the amoxicillin MIC against *H. pylori* while preserving *L. casei* viability with inulin	[31]
Mucus/biofilm persistence	PEGylated lipid–polymer NPs with chitosan core	Clarithromycin + rhamnolipid	Mucus penetration and biofilm disruption	Biofilm and mucus models	Improved mucus penetration and biofilm eradication	[17]
Biofilm persistence and recolonization	Self-assembled nanodrugs	Berberine derivatives + rhamnolipids	Mucus penetration, EPS disruption, anti-readhesion	*in vitro* and infected mice	Reduced biofilm, bacterial burden and inflammatory injury	[39]
Drug-resistant *H. pylori*	Cerium oxide responsive nanozyme	Catalytic CeO_2_ platform	Bacterial killing plus ROS modulation	*in vitro*/infection models	Bactericidal ROS generation with epithelial ROS scavenging	[43]
Persistent *H. pylori*/mucosal injury	Chitosan-coated lipid/copper-organic framework	Rhamnolipid + phosphatidic acid + Cu^2+^	Biofilm dispersion, intracellular clearance, autophagy, repair	*in vitro* and infected animals	Clearance of multiple bacterial reservoirs with reduced inflammation and preservation of the microbiota	[18]
*H. pylori*/host-directed therapy	Chitosan microparticles (not nanoscale)	*M. bovis* BCG	Compartment-adapted delivery and immunomodulation	*Cavia porcellus*	Reduced gastric colonization with mucin and immune-profile modulation	[8,9,10]
Precancerous gastric mucosa	Gastric epithelial cell-localizable oral nanomedicines	Resveratrol + lactoferrin + modified fucoidan	Intracellular *H. pylori* clearance and premalignant-state modulation	Infection/intestinal-metaplasia models	Bacterial clearance and improvement in intestinal metaplasia-associated features	[19]
*H. pylori*-tumor interface	Curcumin-stabilized silver NPs	Curcumin + AgNPs	Combined antibacterial and antiproliferative activity	*H. pylori* and AGS cells	Urease inhibition, antibacterial activity, and reduced AGS-cell proliferation	[59]
HER2-positive GC	HER2-targeted NPs	DM1	Molecularly targeted cytotoxic delivery	NCI-N87 and *in vivo* tumors	Preferential HER2-positive tumor targeting and antitumor activity	[20]
Established GC	Cancer-cell-membrane-coated PLGA NPs + YSA peptide	STM2457	EphA2-oriented delivery and pathway modulation	GC cells and *in vivo* tumors	Suppression of oncogenic signaling and enhancement of the anti-PD-1 response	[49]
Established GC/MDR	PLGA nanoparticles	Docetaxel + LY294002	Co-delivery targeting PI3K/Akt-associated resistance	GC cells; orthotopic and xenograft mouse models	Controlled release, increased apoptosis and enhanced antitumor activity	[21]
Established GC	pH/ROS dual-responsive NPs	Docetaxel	TME-responsive release and tumor penetration	GC spheroids and *in vivo* tumors	Deep spheroid penetration and enhanced tumor inhibition	[34]
GC/immune microenvironment	iRGD-conjugated PLGA NPs	siSOX9 + chlorin e6 + L-arginine	Photodynamic therapy plus immune modulation	Preclinical GC models	Enhanced dendritic-cell maturation and CD8 T-cell activation	[54]
Established GC	Histamine-functionalized PLA NPs	Doxorubicin or 5-fluorouracil	Functional polymer-mediated anticancer delivery	AGS cells; THP-1 Blue monocytes for inflammation-associated readouts	Oxidative stress and DNA damage, apoptosis, mitochondrial and cell-cycle changes, and modulation of inflammatory signaling	[35,46,47]
*H. pylori*-associated GC model	PLA-His-DOX NPs	Doxorubicin	Nanotherapy tested with infection-associated stimuli	3D AGS spheroids + CagA/LPS	Cytotoxicity, DNA damage, and apoptosis, with IL-1β/IL-8 differences not normalized to viable cell number	[22]

Abbreviations: AgNPs, silver nanoparticles; BCG, Bacillus Calmette–Guérin; CagA, cytotoxin-associated gene A protein; CeO_2_, cerium oxide; DOX, doxorubicin; EPS, extracellular polymeric substances; GC, gastric cancer; *H. pylori*, *Helicobacter pylori*; HER2, human epidermal growth factor receptor 2; LPS, lipopolysaccharide; MDR, multidrug resistance; NPs, nanoparticles; PD-1, programmed cell death protein 1; PLA, poly(lactic acid); PLGA, poly(lactic-co-glycolic acid); ROS, reactive oxygen species; TME, tumor microenvironment; 5-FU, 5-fluorouracil.

## Data Availability

No new data were created or analyzed in this study. Data sharing is not applicable to this article.

## Abbreviations

The following abbreviations are used in this manuscript:

*H. pylori*	*Helicobacter pylori*
GC	gastric cancer
MALT	mucosa-associated lymphoid tissue
RANTES	Regulated upon Activation, Normal T cell Expressed and Secreted
NF-κB	nuclear factor kappa B
JAK-STAT	Janus kinase-signal transducer and activator of transcription
MAPK	mitogen-activated protein kinase
PI3K/Akt	phosphoinositide 3-kinase/protein kinase B
PEG	polyethylene glycol
*M. bovis*	*Mycobacterium bovis*
BCG	Bacillus Calmette–Guérin
GlcNAc	N-acetyl-D-glucosamine
ECM	extracellular matrix
ROS	reactive oxygen species
CagA	cytotoxin-associated gene A
VacA	vacuolating cytotoxin A
STAT3	signal transducer and activator of transcription 3
LPS	Lipopolysaccharide
IL	Interleukin
CAF	cancer-associated fibroblast
PLA	poly(lactic acid)
PLGA	poly(lactic-co-glycolic acid)
EPR	enhanced permeability and retention
HER2	human epidermal growth factor receptor 2
PD-L1	Programmed Death-Ligand 1
HIF-1α	hypoxia-inducible factor 1 alpha
MDR	multidrug resistance
nab-paclitaxel	nanoparticle albumin-bound paclitaxel
TNF-α	tumor necrosis factor alpha
AgNPs	silver nanoparticles
CeO_2_	cerium oxide
DOX	Doxorubicin
EPS	extracellular polymeric substances
His	Histamine
NPs	Nanoparticles
TME	tumor microenvironment
5-FU	5-fluorouracil
EPIYA	glutamic acid–proline–isoleucine–tyrosine–alanine
SHP2	Src homology 2 domain-containing protein tyrosine phosphatase 2
ERK	extracellular signal-regulated kinase
SIRT1	sirtuin 1
ZO-1	zonula occludens-1
ICAM-1	intercellular adhesion molecule 1
RNS	reactive nitrogen species
EphA2	ephrin type-A receptor 2
siRNA	small interfering RNA
